# Discriminatory Capacity of Anthropometric Indicators and Physical Fitness Tests for Identifying Waist-to-Height Ratio-Defined Cardiometabolic Risk in Adolescents According to Sex and Biological Maturity

**DOI:** 10.3390/children13091201

**Published:** 2026-09-05

**Authors:** Victoria López-Lombó, Adrián Mateo-Orcajada, Lucía Abenza-Cano, J. Arturo Abraldes, Mario Albaladejo-Saura, Raquel Vaquero-Cristóbal

**Affiliations:** 1 Research Group Movement Sciences and Sport (MS&SPORT), Department of Physical Activity and Sport Sciences, Faculty of Sport Sciences, University of Murcia, 30720 San Javier, Murcia, Spain; victoria.lopezl@um.es (V.L.-L.); abraldes@um.es (J.A.A.); raquel.vaquero@um.es (R.V.-C.); 2 Facultad de Deporte, UCAM Universidad Católica San Antonio de Murcia, 30107 Guadalupe, Murcia, Spain; labenza@ucam.edu (L.A.-C.); mdalbaladejosaura@ucam.edu (M.A.-S.)

**Keywords:** adolescents, body composition, maturity status, physical fitness, ROC curves, sex, WHtR-defined cardiometabolic risk, Youden index

## Abstract

**Highlights:**

**What are the main findings?**

- Anthropometric parameters (BMI, fat mass percentage, skinfolds, and hips girth) demonstrated high diagnostic accuracy for identifying WHtR-defined surrogate risk across sex and biological maturation strata.
- Unadjusted physical fitness tests showed poor overall discriminatory capacity, whereas normalizing performance metrics for body mass (e.g., relative CMJ and VO_2_ max) improved classification utility.
- An observed pattern of descending optimal cut-off thresholds for anthropometric indicators was evident across progressively later-maturing cohorts.

**What are the implications of the main findings?**

- Biological maturation timing should be carefully considered when future research derives and externally validates adolescent health screening thresholds, as current thresholds remain exploratory and sample-specific.
- Physical fitness batteries in educational settings should primarily serve to track functional and motor development; when evaluated in relation to adiposity-defined risk, performance metrics must be normalized for body mass.

**Abstract:**

Background/Objectives: Field-based health screening in adolescents requires accurate, non-invasive, and feasible diagnostic tools. This study evaluated the discriminatory capacity of anthropometric parameters and physical fitness tests for identifying waist-to-height ratio (WHtR)-defined surrogate cardiometabolic risk across sex and biological maturity status, determining exploratory cut-off thresholds. Methods: A cross-sectional study was conducted with 2944 Spanish adolescents (1459 males, 1485 females; age: 13.35 ± 1.20 years). Anthropometric variables (BMI, skinfolds, fat/muscle mass) and physical fitness tests (20-m shuttle run, handgrip, CMJ, SBJ, 20-m sprint, curl-up; evaluated in raw and body-mass-normalized formats) were assessed. Biological maturation was estimated via Age at Peak Height Velocity (APHV) using Mirwald equations and categorized into early, on-time, and late maturers relative to the sample mean. Surrogate risk was operationalized strictly as WHtR ≥ 0.50, without direct assessment of biochemical or hemodynamic parameters. Receiver operating characteristic (ROC) curves, optimal Youden-derived cut-offs, and internal bootstrap validation (1000 resamples) were performed. Results: Anthropometric parameters demonstrated high discriminatory performance for detecting WHtR-defined surrogate risk across all sex and maturation cohorts (AUC = 0.88–0.98). Unadjusted physical fitness tests showed poor overall classification performance. However, normalizing fitness metrics for body mass (particularly relative CMJ and cardiorespiratory fitness) substantially improved discriminatory capacity. Optimal cut-off values for anthropometric indicators displayed an observed descending pattern across progressively later-maturing cohorts. Conclusions: Anthropometric parameters represent highly accurate field proxies for WHtR-defined surrogate risk screening in adolescents. While unadjusted fitness metrics have limited utility for this anthropometric outcome, body-mass-normalized fitness parameters substantially recover classification capacity. Biological maturation timing should be carefully considered when future research derives and externally validates adolescent screening thresholds, as current cut-offs remain exploratory and sample-derived.

## 1. Introduction

Childhood obesity constitutes one of the main public health problems worldwide. According to the World Health Organization (WHO), more than 390 million children and adolescents aged 5 to 19 years are overweight, of whom more than 160 million live with obesity, a figure that has quadrupled since the end of the 20th century [1,2]. This condition impacts various organs and systems in children and adolescents and tends to persist into adulthood [3,4], thereby increasing the risk of developing non-communicable chronic diseases such as Type 2 diabetes mellitus, various cardiovascular diseases, and metabolic liver disease, among others [5,6]. In addressing these issues, a clear conceptual distinction must be maintained throughout between general obesity identification, actual clinical cardiometabolic risk (requiring direct assessment of blood pressure, glycemic control, and lipid profiles) [7], and anthropometrically derived surrogate risk indicators, which are useful for screening or stratification but do not directly diagnose metabolic disease [8,9].

Obesity affects body composition and physical fitness in adolescents. Previous research has shown that adolescents with obesity exhibit a significant increase in both subcutaneous and visceral adiposity, accompanied by greater fat-free mass and higher absolute muscle development [10], particularly in the lower limbs, as an adaptation to the mechanical overload derived from excess body weight [11]. Consequently, numerous studies have confirmed that this population tends to display higher values of absolute strength and power compared to their normal-weight peers [11,12]. However, this increase in muscle mass does not compensate for the excess adipose tissue, leading to a decline in relative strength and power [13,14], as well as poorer performance in functional tasks requiring propulsion of one’s own body weight, including speed, agility, jumping, and cardiorespiratory fitness tests [14]. Therefore, scientific evidence suggests that, despite demonstrating a greater capacity to produce strength in absolute terms, adolescents with obesity experience a deterioration in overall physical performance due to an unfavorable strength-to-weight ratio and lower functional quality of muscle tissue [13].

In light of the negative impact of obesity on adolescent health, public health strategies have prioritized the prevention and early detection of elevated central adiposity and associated cardiometabolic risks, leading to the design and implementation of valid, cost-effective, and easy-to-apply population screening tools in real-world settings [15,16,17]. Among these settings, the school environment has been one of the most extensively analyzed, as it constitutes an ideal scenario due to its high population coverage and the feasibility of implementing standardized field assessments [16].

Among the most widely used tools for assessing the health status of children and adolescents are the analysis of body composition via anthropometric parameters and physical fitness testing [18,19]. Anthropometric measurements allow for the estimation of adipose tissue accumulation and distribution, as well as musculoskeletal development in children and adolescents [20]; meanwhile, physical fitness tests, particularly those assessing cardiorespiratory capacity and strength production, represent key health status markers [14].

These evaluations are of great relevance, as they have traditionally been used for descriptive or monitoring purposes, demonstrating a consistent bidirectional relationship among obesity, body composition alterations, and physical fitness impairment [13,16]. However, the application of these assessments as screening tools aimed at the early identification of anthropometrically defined risk has been less frequent. In fact, few studies have directly compared the discriminatory capacity of anthropometric indicators and physical fitness tests to identify adolescents at higher risk of presenting central adiposity-related alterations [21,22]. This limitation hinders decision-making in the school context, where it is necessary to determine which techniques, tests, and instruments offer greater validity and reliability to detect health risks quickly and at minimal cost.

Furthermore, although available evidence has proposed reference values for certain anthropometric and physical fitness variables, most studies have been conducted for normative purposes rather than as tools establishing cut-off thresholds for early screening in educational and clinical settings [15]. While a clustered cardiometabolic-risk score combining blood pressure and biochemical markers represents the clinical reference standard, its implementation in mass school-based screening is constrained by invasive procedures, financial costs, and logistical complexity [23]. Consequently, the waist-to-height ratio (WHtR) is a non-invasive, highly feasible surrogate criterion of central adiposity and associated risk [24,25]. Nevertheless, evaluating body mass index (BMI), skinfolds, and body fat percentage against an outcome derived from another anthropometric index is conceptually distinct from validating these measures against independent clinical endpoints (such as blood pressure or blood biochemistry). This study specifically addresses the discriminatory capacity for detecting central adiposity-defined risk rather than direct metabolic dysfunction. Within this context, scientific evidence suggests that certain anthropometric variables, as well as specific physical fitness indicators, could possess relevant discriminatory capacity for identifying adolescents exceeding this surrogate risk threshold [26]. Nevertheless, available findings remain inconsistent and heterogeneous, partly due to differences in the study population, sex, maturity status, and assessment procedures employed [27,28].

In this regard, much of the literature has relied on body mass index (BMI) [26,29], a useful yet limited indicator for individual risk stratification, as it does not differentiate between fat mass and fat-free mass, provides no information on adipose tissue distribution, and can under- or overestimate risk in adolescents with differing growth patterns [30,31]. Conversely, the use of more specific anthropometric variables offers a more precise characterization of excess adiposity and, consequently, a higher capacity to discriminate among adolescents [31]. Furthermore, most studies have focused on isolated variables or specific samples; thus, research simultaneously comparing the discriminatory utility of body composition indicators and physical fitness tests within the same adolescent population remains scarce, particularly in the school setting.

In addition to the lack of prior research addressing this issue, significant differences in body composition and physical fitness are observed between male and female adolescents during adolescence [27,32], demonstrating that sex acts as a highly relevant modulating factor in the changes observed in these variables [18]. Similarly, biological maturity status plays a major role, as distinct profiles determine the rate and extent of development in adolescents’ body composition and physical fitness variables [32,33,34]. When accounting for maturation, maturity timing estimated via peak height velocity age (APHV) using Mirwald equations and categorized using a sample-relative threshold (±0.5 years around the sample mean) reflects relative biological timing, which must be strictly distinguished from direct clinical pubertal staging (such as Tanner stages) or endocrine evaluations [35,36]. For this reason, including both sex and estimated biological maturity timing as differentiating elements is essential when analyzing which variables can serve as discriminators of WHtR-defined risk, although this has been sparsely explored in previous studies.

These gaps in the scientific literature limit the population screening capacity of body composition and physical fitness for detecting early health risks. Therefore, the aim of the present study was to evaluate the discriminatory capacity of anthropometric and physical fitness variables for discriminating adolescents with waist-to-height ratio-defined elevated cardiometabolic risk according to sex and estimated biological maturity status, as well as to define optimal exploratory cut-off points for each variable.

## 2. Materials and Methods

### 2.1. Design

A cross-sectional study was conducted to evaluate the discriminatory capacity of body composition and physical fitness variables for identifying waist-to-height-ratio defined cardiometabolic risk in adolescents, determining optimal exploratory cut-off points according to sex and estimated maturity status.

The research protocol was previously approved by the Institutional Ethics Committee of the Universidad Católica de Murcia (code: CE022102), following the ethical principles established by the World Medical Association and the Declaration of Helsinki. Furthermore, both the design of the present study and the preparation of the manuscript adhered to the recommendations of the STROBE (Strengthening the Reporting of Observational Studies in Epidemiology) guidelines for cross-sectional studies [37].

### 2.2. Participants

Sample selection was conducted through non-probabilistic convenience sampling. Eight compulsory secondary education schools distributed across different areas of the Region of Murcia (Spain) were included, chosen for having a high number of enrolled students. After obtaining institutional authorization, meetings were held with school management teams and Physical Education teachers to present the objectives and procedures of the study. Subsequently, both students and their families were informed about the research. Participation was entirely voluntary and was formalized through the signing of informed consent by the participants and their legal guardians, ensuring the confidentiality and anonymity of the collected information at all times.

A total of 3210 adolescents were initially invited to participate. Of these, 186 were excluded for not obtaining signed informed consent or declining participation, and 80 were excluded for not completing the full battery of physical tests or anthropometric protocols. Thus, the final sample comprised 2944 male and female adolescents (males: *n* = 1459; females: *n* = 1485; mean age: 13.35 ± 1.20 years), representing an overall participation rate of 91.7%. Descriptive statistics for the sample are presented in Table 1.

The sample size was estimated to ensure adequate statistical power for Receiver Operating Characteristic (ROC) curve analyses and area under the curve (AUC) discrimination. Assuming an expected prevalence of WHtR-defined cardiometabolic risk (WHtR ≥ 0.50) of 15–20% in Spanish adolescents [38], a null hypothesis AUC of 0.50 vs. an alternative AUC ≥ 0.70, a significance level of α = 0.05, and a statistical power of 1 − β = 0.90, a minimum required sample of 2735 adolescents across sex and maturation strata was calculated using Rstudio (v3.15.0; RStudio Inc., Boston, MA, USA).

The inclusion criteria were: (a) being between 12 and 16 years of age; (b) presenting no injuries or having undergone surgical interventions that would prevent performance in the physical fitness tests; and (c) being enrolled in compulsory secondary education (ESO). The exclusion criterion was established as failing to fully complete the physical fitness tests or anthropometric measurements.

### 2.3. Instruments

#### 2.3.1. Anthropometric Variables

Anthropometric measurements were performed by Level 3 and Level 4 anthropometrists certified by the International Society for the Advancement of Kinanthropometry (ISAK). The protocol measurements included three basic measures (body mass, height, and sitting height), five girths (arm relaxed, waist, hips, thigh, and calf), and three skinfolds (triceps, thigh, and calf) [39].

All measurements were conducted following the standardized protocols established by ISAK [39]. To ensure evaluation reliability, each variable was measured in duplicate. When the difference between the two measurements exceeded 1% for basic measures and girths, or 5% for skinfolds, a third measurement was taken. In cases where only two records were obtained, the mean value was used for statistical analyses. Conversely, when a third measurement was necessary, the median of the three was used as the final value, according to ISAK recommendations [39].

Height and sitting height were measured using a SECA 213 stadiometer (SECA, Hamburg, Germany) with a precision of 0.1 cm. Body mass was recorded using a TANITA BC 418-MA segmental scale (TANITA, Tokyo, Japan) with a precision of 100 g. Body girths were assessed using a Lufkin W606PM non-extensible anthropometric tape (Lufkin, Missouri City, TX, USA) with a precision of 0.1 cm, whereas skinfolds were measured using a Harpenden skinfold caliper (Burgess Hill, UK) with a precision of 0.2 mm. All instruments were calibrated prior to the start of the measurements.

To check measurement quality, intra- and inter-evaluator technical error of measurement (TEM) was calculated in a subsample. Intra-evaluator TEM was 0.04% for basic measures, 1.18% for skinfolds, and 0.03% for girths. Meanwhile, inter-evaluator TEM reached values of 0.06% for basic measures, 1.99% for skinfolds, and 0.08% for girths.

From the recorded anthropometric variables, BMI (body mass/[height]^2^) [40], WHtR (waist girth/height), fat mass [40], muscle mass [41], corrected girths of the arm [arm relaxed girth − (π × triceps skinfold)], thigh [middle thigh girth − (π × thigh skinfold)], and calf [calf girth − (π × calf skinfold)] [39] and the three skinfolds sum [39] were calculated. To avoid circular reasoning and mathematical incorporation bias (as waist girth is a direct numerator in the reference index), waist girth was utilized strictly to compute WHtR and was excluded as an independent discriminant variable in the ROC curve discriminatory analyses.

WHtR was used to estimate the surrogate cardiometabolic risk of the adolescents. Unlike other anthropometric indices, WHtR takes individual height into account, allowing for a better assessment of abdominal fat distribution and its association with obesity risk factors. To establish the presence or absence of such risk, the cut-off point proposed by Zong et al. [24] for European child and adolescent populations was used, considering a WHtR ≥ 0.50 as an indicator of elevated surrogate cardiometabolic risk and a WHtR < 0.50 as the absence thereof (without assessing direct biochemical or hemodynamic markers). This cut-off value has demonstrated adequate capacity to discriminate the presence of multiple risk factors, constituting a simple, reproducible tool with high discriminatory power for identifying cardiometabolic risk in adolescent populations, regardless of age and sex [24].

Biological maturation timing was estimated from the Age at Peak Height Velocity (APHV), a widely recognized non-invasive surrogate of somatic maturation during adolescence [42]. Maturity offset (estimated years to/from PHV) was calculated using the sex-specific anthropometric prediction equations developed by Mirwald et al. [43], and APHV was obtained by subtracting maturity offset from chronological age (APHV = chronological age—maturity offset). Although these equations offer high field feasibility and reproducibility for large-scale epidemiological studies [33], their predictive accuracy must be interpreted with caution. Anthropometric estimation models carry inherent limitations, including systematic estimation errors and lower precision when evaluating individuals who are chronologically distant from their actual PHV or located at the extreme ends of the maturation spectrum [44,45].

Finally, participants were stratified into three maturation cohorts using a ±0.5-year threshold relative to the sex-specific sample mean APHV [46]: early maturation (<−0.5 years relative to sample mean), on-time maturation (±0.5 years), and late maturation (>+0.5 years relative to sample mean). This ±0.5-year cutoff around the sample mean was selected to establish balanced operational cohorts for internal comparative analyses [47]. However, because this categorization is calculated strictly relative to the internal sample distribution, it represents sample-dependent relative maturity timing rather than absolute clinical pubertal status. Consequently, an adolescent classified as “early” within this study is early relative to this specific sample and would not necessarily be classified as early relative to an external normative population or through direct clinical pubertal staging (such as Tanner stages).

For males, the sample mean APHV was 13.82 years, yielding the following APHV classification boundaries: early maturers (APHV < 13.32 years), on-time maturers (APHV between 13.32 and 14.32 years), and late maturers (APHV > 14.32 years). For females, the sample mean APHV was 12.58 years, resulting in classification boundaries of: early maturers (APHV < 12.08 years), on-time maturers (APHV between 12.08 and 13.08 years), and late maturers (APHV > 13.08 years). Additionally, the mean chronological age at assessment for each group was as follows: early males (13.92 years old), on-time males (14.38 years old), late males (15.01 years old), early females (13.02 years old), on-time females (14.33 years old) and late-females (16.16 years old).

#### 2.3.2. Physical Fitness Test

Participants’ physical fitness was assessed through a battery of validated tests for adolescent populations, designed to evaluate different components of physical performance, including cardiorespiratory fitness, upper-limb muscle strength, lower-limb power, running speed, and muscular endurance, following standardized protocols used in previous studies [19,48] and using previously calibrated instruments.

Cardiorespiratory fitness was evaluated via the 20-m shuttle run test, a maximal incremental test widely validated and displaying high reliability for application in adolescent populations [49]. During the assessment, participants had to repeatedly cover a distance of 20 m, synchronizing their running pace with acoustic signals emitted by a recording. Initial speed progressively increased at each level of the protocol, requiring an increase in running pace. The test ended when the participant reached exhaustion or was unable to reach the 20-m line before the acoustic signal on two consecutive occasions. The last completed level was used to estimate maximal oxygen uptake (VO_2_ max) using the equation proposed by Léger et al. [49].

Upper-limb strength was assessed through handgrip strength. This was determined using a Takei TKK5401 portable digital dynamometer (Takei Scientific Instruments, Tokyo, Japan) [50]. Participants performed the test unilaterally with both hands separately, applying maximal grip force with the elbow fully extended throughout the execution [51]. Both absolute handgrip strength (kg, recorded as the maximum value between hands) and relative handgrip strength (kg × kg^−1^ body mass) were calculated and included in the analyses to account for body mass confounding.

Lower-limb explosive strength and power were evaluated using the Countermovement Jump (CMJ) and Standing Broad Jump (SBJ). For the CMJ, participants performed the jump on a force platform (MuscleLab, Stathelle, Norway) with a sampling frequency of 200 Hz, recording the maximum height achieved during flight. The test started from an upright position with hands resting on the hips. Subsequently, participants performed a knee flexion of approximately 90°, followed by an explosive extension of the lower limbs at maximal velocity. Throughout the execution, hands were kept on the hips, the trunk in a vertical position, and the knees and ankles fully extended [52]. To account for body mass and stature confounding, CMJ performance was evaluated as both absolute jump height and relative jump height normalized to body mass (cm × kg^−1^). For the SBJ, participants stood behind the starting line with feet parallel and shoulder-width apart. From this position, they performed a maximal horizontal jump using a preparatory movement with knee flexion and free arm swing to assist propulsion. The distance achieved was measured from the starting line to the most rearward heel at the moment of landing. Attempts were considered valid only when the participant maintained balance upon landing, without touching the floor with their hands or taking steps backward [53]. To eliminate the confounding influence of individual height, SBJ performance was evaluated as both absolute raw distance (m) and relative distance normalized to height (m × m^−1^ height).

Speed was evaluated using a maximal 20-m sprint. Participants started the test from a static position behind the starting line, freely deciding when to begin the sprint [54]. The time taken to cover the distance was recorded using a single-beam photocell system (Polifemo Light, Microgate, Italy) placed at hip height. This photocell placement has been shown to improve measurement reliability, as it reduces the likelihood of the beam being broken by arm movements before the trunk, with an estimated probability of approximately 4%, compared to 60% observed when devices are placed at chest height [55,56].

Finally, abdominal muscular endurance was assessed using the curl-up test. Participants lay in a supine position with knees flexed at 90°, feet flat on the floor, and arms crossed over the chest. They then performed the maximum number of trunk flexions possible within one minute or until exhaustion. Repetitions were considered valid when the upper back lifted off the floor [57].

### 2.4. Procedure

To reduce the influence of potential confounding variables, all assessments were conducted under standardized conditions. Anthropometric measurements were carried out in the sports hall changing rooms, maintaining a stable temperature, and were always performed between 8:30 a.m. and 2:30 p.m., coinciding with regular Physical Education class schedules, so that each group was evaluated within the same time slot. Physical fitness tests were conducted in an indoor sports hall to prevent weather conditions from affecting performance. Furthermore, all measurements for a specific test were supervised by the same expert researcher to ensure maximum standardization and minimize inter-evaluator variability.

Evaluations commenced with the anthropometric assessment of the participants. Subsequently, the procedures for the physical fitness tests were explained to the adolescents, and a familiarization phase was conducted for the handgrip, CMJ, SBJ, 20-m sprint, and curl-up tests to ensure proper execution of each test. Following this, participants completed a standardized warm-up consisting of low-intensity continuous running and specific joint mobility exercises for the involved muscle groups.

Two trials were performed for each physical fitness test, with the best score recorded as the final result. A two-minute recovery period was established between trials of the same test, while a five-minute rest period was observed between different tests. The order of test execution was randomized for each participant to minimize any potential order effect on performance. The 20-m shuttle run test was administered at the end of the physical fitness battery due to its maximal incremental nature and the high level of fatigue it generates, which could compromise performance in the remaining tests. The assessment protocol was designed in accordance with the recommendations of the National Strength and Conditioning Association (NSCA) [58], considering metabolic demands, test-induced fatigue, and required recovery times.

### 2.5. Data Analysis

Statistical data were analyzed using SPSS statistical software (Version 25.0; SPSS Inc., Chicago, IL, USA). Continuous variables were presented as mean and standard deviation (M ± SD). Prevalence of elevated WHtR-defined cardiometabolic risk was calculated as absolute frequencies (n) and percentages (%) for each sex and maturity cohort. Thus, among early males, 69 were at risk (31.08%), among on-time males 159 (15.76%), and among late males 53 (23.25%); while among early females, 57 were at risk (24.26%), among on-time females 167 (16.49%), and among late females 47 (19.83%).

Normality of data distribution was verified using the Kolmogorov–Smirnov test combined with visual inspection of Q-Q plots and skewness/kurtosis analysis due to the large sample size (*n* = 2944). To evaluate differences in anthropometric, body composition, and physical fitness characteristics across the three biological maturation cohorts (early, on-time, and late), a one-way analysis of variance (ANOVA) was performed, executed independently for males and females, followed by Bonferroni post hoc tests for pairwise comparisons. Effect size was determined using the partial eta squared parameter ($\eta_{p}^{2}$), categorized as small ($\eta_{p}^{2}$ > 0.01), medium ($\eta_{p}^{2}$ > 0.06) or large ($\eta_{p}^{2}$ > 0.14).

To determine the discriminatory capacity of anthropometric and physical fitness variables in identifying adolescents with WHtR-defined risk (WHtR ≥ 0.50), receiver operating characteristic (ROC) curve analyses were performed. The area under the curve (AUC) was calculated along with its respective 95% confidence intervals (95% CI). To ensure consistent interpretation of diagnostic accuracy across all metrics, the directionality of performance tests where lower scores indicate higher risk (e.g., cardiorespiratory fitness, jump distance, muscle strength, and curl-ups) was standardized by calculating 1–AUC. This transformation aligns all variables so that lower physical performance reflects higher cardiometabolic risk, allowing AUC values to be interpreted uniformly relative to the line of non-discrimination (0.50). The discriminatory capacity based on AUC values was interpreted according to Hosmer and Lemeshow criteria [59]: excellent (>0.90), good (0.80–0.90), moderate (0.70–0.80), and poor or null (<0.70).

Optimal cut-off points for each variable were established by maximizing Youden’s index (J = Sensitivity + Specificity − 1), which reflects the point on the ROC curve where the vertical distance to the line of non-discrimination (chance) is maximized, weighting true positive and true negative rates equally. Youden’s index values range between 0 and 1, where values closer to 1 indicate greater diagnostic capacity and values closer to 0 indicate weaker capacity [60,61]. From the optimal cut-off point identified by this index, corresponding sensitivity and specificity values were reported. To assess internal estimate stability and potential optimism/overfitting, an internal validation procedure was performed using a non-parametric bootstrap resampling method (1000 resamples). Bias-corrected and accelerated (BCa) 95% confidence intervals (95% CI), standard errors (SE), and bootstrap bias estimates were calculated for the Area Under the Curve (AUC), optimal cut-off values, sensitivity, specificity and the Youden index across sex and maturity status strata. For all statistical analyses, the alpha significance level was set a priori at *p* < 0.05.

## 3. Results

### 3.1. Descriptive Characteristics and Maturation-Based Differences

Table 1 presents the descriptive characteristics of the full sample and comparisons among biological maturation groups stratified by sex. Overall, the prevalence of WHtR-defined cardiometabolic risk was highest in early maturers for both males (31.08%) and females (24.26%). In males, ANOVA revealed statistically significant differences (*p* < 0.001) across all anthropometric and body composition parameters, with early-maturing males displaying significantly higher body mass, BMI, girths, muscle mass, fat mass, and skinfold thickness compared to on-time and late maturers. Regarding absolute physical fitness in males, only VO_2_ max (*p* = 0.015) showed significant differences among absolute tests, with higher values in early and on-time maturers compared to late maturers. However, when normalized for body mass, relative handgrip strength, and relative CMJ differed significantly, with late maturers displaying higher relative performance. In females, a consistent pattern was observed for anthropometry and body composition, with early-maturing females showing significantly higher values across all variables (*p* < 0.001). For female physical fitness, significant differences were found in VO_2_ max, CMJ, curl-up and SBJ, as well as across all relative fitness indicators, with early and on-time maturers performing higher than late ones (*p* < 0.001).

**Table 1:** Differences in the study variables among the different maturation groups in males and females.

Variables	Full Sample (*n* = 2944)	Males						Females					
		Early (*n* = 222)	On Time (*n* = 1009)	Late (*n* = 228)	F	*p*	$\eta_{p}^{2}$	Early (*n* = 235)	On Time (*n* = 1013)	Late (*n* = 237)	F	*p*	$\eta_{p}^{2}$
At WHtR Risk, *n* (%)	552 (18.75)	69 (31.08)	159 (15.76)	53 (23.25)	-	-	-	57 (24.26)	167 (16.49)	47 (19.83)	-	-	-
Body mass (kg)	57.10 ± 13.73	73.61 ± 17.45	58.54 ± 12.34	51.63 ± 11.92	66.13	<0.001	0.19	62.16 ± 15.98	53.11 ± 11.45	53.27 ± 8.57	39.19	<0.001	0.11
BMI (kg/m^2^)	21.26 ± 3.96	24.09 ± 5.16	20.99 ± 3.63	19.76 ± 3.62	26.96	<0.001	0.09	22.72 ± 4.27	20.83 ± 3.69	21.68 ± 3.57	21.78	<0.001	0.06
Hips girth (cm)	91.01 ± 9.37	98.12 ± 11.06	89.70 ± 8.64	85.72 ± 8.75	43.65	<0.001	0.14	95.74 ± 9.07	90.71 ± 8.61	92.18 ± 7.79	24.61	<0.001	0.07
Corr. arm girth (cm)	21.38 ± 3.47	23.68 ± 4.61	22.33 ± 3.46	21.08 ± 3.42	14.63	<0.001	0.05	21.31 ± 2.65	20.27 ± 2.86	20.33 ± 2.93	8.28	<0.001	0.03
Corr. thigh girth (cm)	40.33 ± 6.04	44.38 ± 7.95	41.74 ± 6.22	39.57 ± 5.15	20.61	<0.001	0.07	40.61 ± 5.12	38.66 ± 4.69	38.46 ± 5.95	9.65	<0.001	0.03
Corr. calf girth (cm)	29.37 ± 3.38	31.52 ± 4.95	30.50 ± 3.05	28.93 ± 3.33	15.04	<0.001	0.05	28.80 ± 2.35	28.32 ± 2.75	28.02 ± 3.84	8.36	0.035	0.01
Fat mass (%)	22.63 ± 10.37	25.24 ± 14.70	18.78 ± 9.80	18.11 ± 10.85	14.70	<0.001	0.05	28.80 ± 9.35	24.59 ± 8.15	26.96 ± 9.04	20.71	<0.001	0.06
Muscle mass (kg)	19.09 ± 5.25	24.95 ± 5.72	21.95 ± 4.74	19.70 ± 4.53	22.75	<0.001	0.08	17.42 ± 3.44	16.01 ± 3.51	15.95 ± 2.97	12.06	<0.001	0.02
Triceps skinfold (mm)	14.69 ± 7.11	16.20 ± 8.77	12.07 ± 6.48	11.88 ± 7.05	32.41	<0.001	0.04	18.86 ± 7.19	15.87 ± 6.20	18.08 ± 6.56	27.03	<0.001	0.04
Thigh skinfold (mm)	21.59 ± 10.78	22.93 ± 14.00	16.93 ± 9.55	16.18 ± 7.98	33.14	<0.001	0.05	27.50 ± 10.70	24.43 ± 9.42	27.56 ± 9.48	15.90	<0.001	0.02
Calf skinfold (mm)	14.99 ± 8.56	17.42 ± 11.44	12.24 ± 7.28	11.40 ± 7.98	39.80	<0.001	0.05	19.96 ± 8.69	16.19 ± 7.64	18.07 ± 9.58	21.69	<0.001	0.03
Sum of 3 skinfolds (mm)	52.82 ± 25.06	57.69 ± 32.52	42.73 ± 22.25	40.99 ± 21.56	16.69	<0.001	0.06	67.57 ± 25.59	58.68 ± 21.35	64.30 ± 23.02	15.58	<0.001	0.05
VO_2_ max (ml/kg/min)	37.02 ± 6.99	39.93 ± 6.27	39.86 ± 7.26	37.38 ± 7.40	41.22	0.015	0.02	36.38 ± 3.96	35.32 ± 4.74	28.85 ± 5.86	143.18	<0.001	0.30
Handgrip right (kg)	26.27 ± 8.78	31.77 ± 10.00	29.26 ± 9.56	26.77 ± 8.88	1.14	0.319	0.00	24.50 ± 5.72	23.54 ± 6.42	23.94 ± 5.85	0.15	0.863	0.00
Relative Handgrip right (kg × kg^−1^)	0.47 ± 0.15	0.41 ± 0.14	0.49 ± 0.15	0.55 ± 0.17	22.24	<0.001	0.07	0.42 ± 0.13	0.49 ± 0.16	0.48 ± 0.12	12.77	<0.001	0.04
Handgrip left (kg)	24.37 ± 8.28	29.97 ± 9.00	27.35 ± 8.92	24.81 ± 8.61	2.61	0.074	0.01	23.15 ± 5.35	21.59 ± 6.04	21.59 ± 5.47	1.08	0.340	0.00
Relative Handgrip left (kg × kg^−1^)	0.43 ± 0.14	0.39 ± 0.13	0.46 ± 0.14	0.51 ± 0.15	17.04	<0.001	0.05	0.39 ± 0.11	0.45 ± 0.15	0.43 ± 0.11	10.32	<0.001	0.03
CMJ (cm)	23.24 ± 7.89	29.62 ± 7.32	27.13 ± 8.07	24.82 ± 7.78	0.51	0.601	0.00	20.36 ± 5.33	21.42 ± 6.21	19.36 ± 6.90	14.98	0.007	0.02
Relative CMJ (cm × kg^−1^)	0.43 ± 0.16	0.35 ± 0.13	0.44 ± 0.16	0.51 ± 0.16	27.16	<0.001	0.08	0.34 ± 0.13	0.45 ± 0.15	0.41 ± 0.14	24.23	<0.001	0.07
20-m sprint (s)	3.80 ± 0.86	3.75 ± 0.62	3.65 ± 0.75	3.76 ± 0.60	0.42	0.658	0.00	4.12 ± 0.65	3.97 ± 0.74	3.94 ± 0.99	2.38	0.094	0.01
Curl up (reps)	27.71 ± 13.08	31.05 ± 12.58	29.92 ± 12.74	28.72 ± 12.46	2.11	0.122	0.01	23.84 ± 12.75	25.59 ± 13.43	27.85 ± 11.77	9.07	<0.001	0.03
SBJ (m)	1.41 ± 0.40	1.62 ± 0.37	1.57 ± 0.39	1.50 ± 0.37	0.29	0.747	0.00	1.31 ± 0.31	1.38 ± 0.32	1.20 ± 0.36	9.55	<0.001	0.03
Relative SBJ (m × m^−1^)	0.86 ± 0.24	0.88 ± 0.20	0.90 ± 0.23	0.93 ± 0.23	1.64	0.194	0.01	0.80 ± 0.19	0.88 ± 0.20	0.80 ± 0.23	12.02	<0.001	0.03

BMI: body mass index; Corr: corrected; VO_2_ max: maximal oxygen consumption; CMJ: countermovement jump; SBJ: standing broad jump.

### 3.2. Diagnostic Performance of Study Variables in Early Maturers

Table 2 summarizes the diagnostic performance of anthropometric, body composition, and physical fitness parameters for identifying WHtR-defined surrogate cardiometabolic risk in early-maturing adolescents. Overall, indicators of overall adiposity and body fat distribution demonstrated the highest classification accuracy in both sexes. Specifically, BMI, percentage of fat mass, sum of skinfolds, and hip girth exhibited outstanding discriminatory capacity (AUC > 0.93), whereas muscle-centric variables (e.g., muscle mass, corrected calf girth) showed markedly lower diagnostic utility. Regarding physical fitness, absolute muscle strength (handgrip) lacked diagnostic value (AUC < 0.56). However, adjusting physical fitness parameters for body mass substantially enhanced their discriminatory capacity; relative CMJ and cardiorespiratory fitness emerged as the most robust fitness-derived discriminators of WHtR-defined surrogate cardiometabolic risk in early-maturing males and females.

### 3.3. Diagnostic Performance of Study Variables in On-Time Maturers

Table 3 displays the diagnostic performance of the evaluated parameters for classifying WHtR-defined surrogate cardiometabolic risk in on-time maturing adolescents. Consistent with the pattern observed in early maturers, measures of overall and regional adiposity, specifically BMI, fat mass percentage, hip girth, and individual skinfold thicknesses, demonstrated outstanding discriminatory capacity in both sexes (AUC > 0.89). Conversely, muscle-centric parameters (e.g., muscle mass, corrected calf girth) provided lower classification utility. Regarding physical fitness, absolute upper-body muscle strength (handgrip) showed low discriminatory values (AUC < 0.59). However, adjusting fitness metrics for body size consistently improved classification performance, with relative CMJ emerging as the most effective fitness-derived discriminator of WHtR-defined surrogate cardiometabolic risk in both males and females.

### 3.4. Diagnostic Performance of Study Variables in Late Maturers

Table 4 summarizes the diagnostic performance of the evaluated parameters for classifying WHtR-defined surrogate cardiometabolic risk in late-maturing adolescents. Indicators of overall and central adiposity, most notably BMI, hip girth, and fat mass percentage, maintained superior discriminatory capacity across both sexes (AUC > 0.88). In contrast, physical fitness parameters generally exhibited lower overall classification accuracy in late maturers compared to earlier maturing cohorts. Absolute muscular strength, sprint, and jump performance displayed poor discriminatory utility. Nevertheless, adjusting performance metrics for body mass systematically recovered discriminatory power, with relative CMJ (AUC up to 0.77) and cardiorespiratory fitness remaining the most viable fitness-derived discriminators of WHtR-defined surrogate cardiometabolic risk in males.

### 3.5. Internal Validation and Bootstrap Model Stability

To assess the internal validity, stability, and potential optimism of the estimates for classifying WHtR-defined cardiometabolic risk, a non-parametric bootstrap procedure with 1000 resamples was performed independently across biological maturation categories (Supplementary Tables S1–S3). Overall, the internal validation demonstrated high stability and minimal bias across all maturation stages for both males and females. In early maturers (Supplementary Table S1), bias values remained consistently low across anthropometric indicators, body composition parameters, and physical fitness components, with standard errors (SE) reflecting precise bootstrap estimations within this sample. Similarly, on-time maturers (Supplementary Table S2) exhibited narrow bias-corrected and accelerated (BCa) 95% confidence intervals, indicating low optimism and consistency in the point estimates. Finally, late maturers (Supplementary Table S3) showed robust parameter estimations in both sexes, with bootstrap estimates aligning closely with the original sample point estimates and acceptable SEs. Collectively, these bootstrap procedures support the internal stability and precision of the ROC-derived estimates across early, on-time, and late maturation groups within the study population.

### 3.6. Graphical Representation of ROC Curves Across Maturation Groups

**Figure 1:**
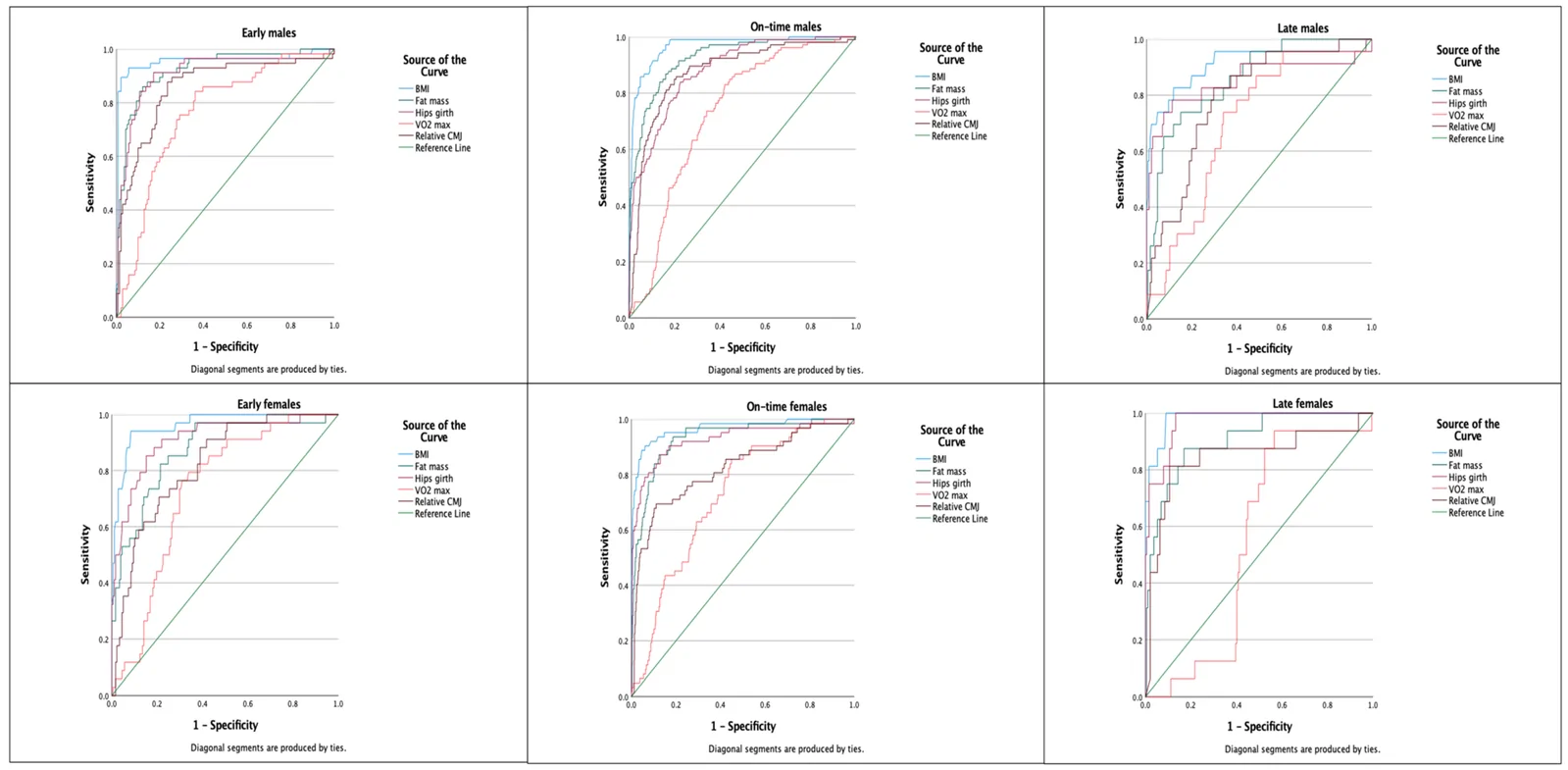
Receiver operating characteristic (ROC) curves of primary candidate parameters for classifying waist-to-height ratio (WHtR)-defined cardiometabolic risk across biological maturation groups by sex. Notes: ROC curves are presented for the primary representative discriminators. Panel A: Males; Panel B: Females. Panels are stratified by biological maturation status (Early, On-time, and Late maturers). BMI: body mass index; CMJ: countermovement jump; VO_2_ max: maximal oxygen consumption.

To enhance visual clarity and avoid curve overlap, Figure 1 illustrates the ROC trajectories for the primary candidate discriminators across biological maturation groups and sexes: BMI, fat mass percentage, hips girth, VO_2_ max and relative CMJ. Visually, the curves confirm the superior classification performance of overall adiposity and body fat distribution metrics, which consistently shifted toward the upper-left corner of the ROC space across all maturation cohorts. Among the physical fitness indicators, relative CMJ and VO_2_ max exhibited the most pronounced discriminatory profiles, outperforming absolute strength and power measures. The complete set of ROC curves for all 22 evaluated anthropometric, body composition, and physical fitness variables is provided in Supplementary Figure S1.

## 4. Discussion

### 4.1. Differences According to Maturity Status and Sex

The objective of the present study was to evaluate the discriminatory capacity of anthropometric and physical fitness variables for identifying adolescents with WHtR-defined surrogate cardiometabolic risk according to sex and estimated maturity status, as well as to define exploratory cut-off points for each variable. Results showed significant differences in body composition and physical fitness variables across biological maturity statuses in both male and female adolescents. Early maturers presented higher average values in body mass, BMI, and parameters related to muscle mass and adiposity. Regarding physical fitness, associations were less uniform: early-maturing males displayed higher values in cardiorespiratory capacity, whereas in females and late-maturing cohorts, performance patterns varied depending on whether fitness was expressed in absolute or body-mass-relative terms. These findings align with prior scientific evidence observing biological maturation as an important factor associated with inter-individual morphological variability during adolescence [43,62]. Observational studies have shown that adolescents reaching puberty earlier tend to display accelerated somatic growth accompanied by greater fat-free mass and higher adipose tissue accumulation compared to on-time or late-maturing peers [27,63]. Conversely, the relationship between maturation and physical performance is non-linear [64], as test performance is associated not only with structural growth [65], but also with unmeasured behavioral and lifestyle factors such as habitual physical activity levels, participation in organized sports, and motor competence [64,65].

These observations support the methodological decision to analyze the discriminatory capacity of these variables stratified by maturity status and sex. Evaluating adolescents strictly by chronological age may obscure biological variation present among individuals at different stages of pubertal development, potentially confounding the observed relationships between these indicators and surrogate cardiometabolic risk [66]. In this sense, biological maturation constitutes a modulating factor that must be considered when evaluating both body composition and physical fitness during adolescence.

### 4.2. Discriminatory Capacity of Study Variables in Early-Maturing Adolescents

In early-maturing adolescents, anthropometric and body composition variables demonstrated high discriminatory capacity for identifying WHtR-defined surrogate risk. Specifically, BMI, percentage of fat mass, sum of skinfolds, and hip girth exhibited the highest diagnostic utility across both sexes. Conversely, muscle-centric parameters (e.g., muscle mass, corrected calf girth) provided lower classification utility. Regarding physical fitness, absolute muscular strength (handgrip) lacked diagnostic utility, whereas adjusting performance for body mass (such as relative CMJ and cardiorespiratory fitness) improved classification capacity in both males and females.

From a methodological standpoint, the primary explanation for the superior diagnostic performance of anthropometric and body composition metrics lies in the nature of the primary outcome itself: because WHtR-defined risk is calculated directly from anthropometric measurements (waist girth and height), other measures of overall and central adiposity (such as BMI, skinfolds, and fat mass) inherently share structural and dimensional variance with the reference standard. Beyond this direct methodological connection, plausible biological factors may also contribute to these observations. During early pubertal development, heightened hormonal activity accelerates adipose tissue accumulation [63], particularly in central depots [67,68], strengthening the cross-sectional association between morphological measures and central adiposity risk. Furthermore, differences observed between sexes suggest that sexual dimorphism inherent to puberty modulates the relationship between physical fitness and cardiometabolic risk from the earliest stages of development [69,70], reinforcing the need to jointly consider sex and maturity status when interpreting these indicators. In addition, the higher discriminatory performance observed when expressing fitness metrics relative to body mass reflects the biomechanical constraint that excess body mass imposes on functional movements requiring body-weight propulsion [71].

### 4.3. Discriminatory Capacity of Study Variables in On-Time Maturing Adolescents

In on-time maturing adolescents, body composition variables, specifically BMI, hip girth, fat mass, and skinfolds, demonstrated high discriminatory capacity for identifying WHtR-defined surrogate risk in both males and females. Among physical fitness metrics, absolute performance tests generally displayed limited discriminatory utility. However, adjusting fitness parameters for body mass, such as relative CMJ, consistently improved classification capacity in both sexes.

The lower classification capacity observed for raw physical fitness metrics compared to anthropometric indicators in this cohort highlights the complex interplay between physical performance and body composition during average pubertal progression. This is a notable finding, given that previous research had shown how cardiorespiratory capacity or lower-limb muscular strength were inversely associated with cardiometabolic risk [72,73]. A plausible explanation, though framed strictly as a hypothesis, given that visceral adiposity and direct metabolic markers were not assessed in the present study, is a potential functional decoupling between neuromuscular performance and central adiposity. In on-time maturing adolescents, performance in strength, power, and cardiorespiratory tests is strongly influenced by unmeasured behavioral and developmental factors, including habitual physical activity patterns, motor competence, and sports participation [74,75], which may operate independently of an individual’s adiposity profile. Consequently, unadjusted physical fitness tests in this group may primarily reflect physical activity exposure and motor skill development rather than central adiposity-defined risk, whereas body-mass-normalized fitness metrics more effectively isolate the functional impairment associated with elevated central adiposity.

### 4.4. Discriminatory Capacity of Study Variables in Late-Maturing Adolescents

In late maturers, overall and regional adiposity indicators (BMI, body fat percentage, skinfolds, and hips girth) maintained high diagnostic utility across sexes. In contrast, unadjusted physical fitness parameters generally exhibited lower overall discriminatory capacity compared to earlier-maturing cohorts, with raw strength, sprint, and jump performances showing poor classification performance. Nevertheless, expressing performance metrics relative to body mass (e.g., relative CMJ and cardiorespiratory fitness) partially recovered discriminatory utility, particularly in males.

The reduced discriminatory performance of raw fitness metrics in late maturers may be explained by the confounding effect that somatic and neuromuscular immaturity exerts on physical test performance [66,76]. Late-maturing adolescents are characterized by delayed structural growth, lower muscle mass, and smaller body dimensions compared to age-matched peers [77], which inherently constrains raw physical performance independently of adiposity levels [62]. Importantly, the poor discriminatory capacity of unadjusted fitness tests against an anthropometric risk threshold in this cross-sectional sample does not establish that physical fitness lacks clinical value for identifying direct biochemical, hemodynamic, or longitudinal cardiometabolic outcomes. Rather, these findings indicate that unadjusted fitness metrics have limited utility as primary screening tools for WHtR-defined risk in late-maturing cohorts, reinforcing the importance of employing adjusted performance metrics and maturation-sensitive evaluation frameworks.

It is important to note that, when interpreting the discriminatory capacity of physical fitness relative to WHtR-defined cardiometabolic risk, point estimates should be considered in conjunction with their 95% confidence intervals, as lower prevalence in this group naturally introduces greater statistical uncertainty.

### 4.5. Comparative Analysis Across Maturity Statuses

Comparative analysis across the three biological maturation stages reveals notable patterns among the evaluated parameters. First, an observed pattern of variation in optimal cut-off points for anthropometric metrics is evident when comparing maturity cohorts, particularly in males. For instance, the exploratory BMI cut-off threshold for identifying WHtR-defined risk shows lower absolute values across progressively later maturing male cohorts (26.15 kg/m^2^ in early maturers, 24.75 kg/m^2^ in on-time maturers, and 22.50 kg/m^2^ in late maturers). In females, optimal cut-off thresholds for BMI exhibit relative cross-sectional stability across cohorts (25.65, 24.65, and 24.78 kg/m^2^ for early, on-time, and late maturers, respectively), while maintaining high diagnostic accuracy (AUC > 0.95).

Second, a clear distinction emerges regarding physical fitness assessments. While unadjusted physical performance tests generally display poor diagnostic accuracy across maturation cohorts, adjusting performance metrics for body mass systematically recovers discriminatory capacity, particularly in males and early/on-time maturers. These findings highlight that individual morphological development, and body dimensions modulate the cross-sectional performance of field tests, emphasizing the relevance of accounting for sex and biological maturity timing when analyzing health-related fitness and body composition in adolescent populations.

### 4.6. Practical Applications

The findings of the present study provide actionable, exploratory insights that may inform future research and assessment protocols in educational and health contexts. Simple, non-invasive anthropometric parameters, specifically BMI, body fat percentage, skinfolds, and hips girth, demonstrate strong cross-sectional performance as practical proxies for identifying WHtR-defined central adiposity risk in adolescents. However, because the cut-off thresholds derived in this study are exploratory and sample-specific, external validation in independent populations must precede any formal implementation in clinical or school-based screening programs. When accounting for pubertal development in field settings, researchers should explicitly specify the assessment methodology used; non-invasive anthropometric prediction equations (such as APHV models) offer high feasibility for field research, though their inherent estimation errors must be acknowledged relative to clinical or radiographic pubertal staging. Finally, physical fitness tests in school settings should primarily serve to monitor motor competence and functional development, as raw performance largely reflects somatic growth and physical activity exposure; when evaluating fitness in relation to adiposity-defined risk, performance metrics must be normalized for body mass to account for body size confounding.

### 4.7. Study Limitations

Despite the results obtained, several limitations must be considered when interpreting the findings. First, the cross-sectional design precludes establishing causal relationships or determining the longitudinal behavior of optimal cut-off thresholds across the pubertal transition. Second, somatic maturation was estimated using APHV prediction equations, which carry inherent estimation errors, particularly at the chronological extremes of adolescence, and reflect sample-dependent relative timing rather than direct clinical pubertal staging (such as Tanner stages) or bone age radiography. Third, cardiometabolic risk was defined strictly using an anthropometric surrogate threshold (WHtR ≥ 0.50). Crucially, because no direct biochemical (e.g., lipid profile, glycemic status) or hemodynamic (e.g., blood pressure) markers were assessed, this study cannot determine whether the proposed cut-offs identify actual clinical metabolic abnormalities. Fourth, although internal model stability was evaluated via bootstrap resampling, the Youden-derived cut-off thresholds lack external validation in an independent sample, which limits their immediate applicability or clinical transferability. Fifth, behavioral factors such as habitual physical activity levels, dietary patterns, and participation in organized sports were not directly measured or incorporated into the statistical models, despite being potential confounders of physical fitness performance. Finally, the sample selection via non-probabilistic convenience sampling within a single geographic region limits the direct generalizability of these specific cut-off thresholds to adolescent populations with different ethnic, socioeconomic, or geographic characteristics.

## 5. Conclusions

In conclusion, anthropometric and body composition parameters, specifically BMI, body fat percentage, skinfolds, and hips girth, demonstrate high cross-sectional discriminatory capacity for identifying WHtR-defined surrogate risk in adolescents across sexes and biological maturation statuses. Conversely, unadjusted physical fitness metrics display poor diagnostic utility due to the confounding effect of body size, although normalizing performance metrics for body mass (such as relative dynamic lower-limb power and cardiorespiratory fitness) partially restores classification performance. Furthermore, the optimal cut-off thresholds identified across biological maturation stages display an observed descending pattern from early to late maturers. Importantly, the cut-off thresholds derived in this study are exploratory and sample-specific and should not be interpreted as immediately transferable clinical standards. Rather than supporting immediate clinical or school-based implementation, these findings demonstrate that biological maturation timing should be carefully considered when future research derives and externally validates screening thresholds for adolescent populations in independent cohorts.

## Figures and Tables

**Table 2:** Optimal cut-off, sensitivity, specificity, Youden index and area under the ROC curves for identifying WHtR-defined surrogate cardiometabolic risk in early-maturing adolescents.

Variables	Males					Females				
	AUC (95% CI)	Cut-Off	Sensitivity	Specificity	Youden	AUC (95% CI)	Cut-Off	Sensitivity	Specificity	Youden
Body mass (kg)	0.86 (0.78, 0.94)	74.67	0.88	0.75	0.63	0.83 (0.76, 0.91)	60.85	0.93	0.66	0.59
BMI (kg/m^2^)	0.99 (0.98, 1.00)	26.15	0.96	0.97	0.93	0.95 (0.92, 0.99)	25.65	0.93	0.90	0.83
Hips girth (cm)	0.93 (0.87, 0.98)	99.50	0.97	0.77	0.73	0.90 (0.82, 0.97)	100.35	0.83	0.83	0.67
Corr. arm girth (cm)	0.70 (0.58, 0.82)	24.45	0.68	0.67	0.35	0.81 (0.71, 0.90)	22.56	0.77	0.79	0.56
Corr. thigh girth (cm)	0.64 (0.51, 0.78)	46.61	0.56	0.73	0.29	0.76 (0.64, 0.87)	44.09	0.67	0.84	0.51
Corr. calf girth (cm)	0.54 (0.41, 0.68)	32.47	0.52	0.63	0.15	0.61 (0.49, 0.73)	30.19	0.50	0.74	0.24
Fat mass (%)	0.95 (0.91, 0.99)	34.49	0.84	0.95	0.79	0.89 (0.83, 0.95)	32.58	0.83	0.80	0.64
Muscle mass (kg)	0.60 (0.46, 0.73)	25.17	0.56	0.60	0.16	0.71 (0.59, 0.84)	19.08	0.67	0.82	0.49
Triceps skinfold (mm)	0.93 (0.88, 0.97)	20.05	0.85	0.93	0.78	0.84 (0.78, 0.91)	21.10	0.84	0.72	0.56
Thigh skinfold (mm)	0.90 (0.86, 0.95)	22.95	0.88	0.81	0.69	0.84 (0.77, 0.92)	33.80	0.78	0.85	0.63
Calf skinfold (mm)	0.92 (0.88, 0.96)	22.90	0.73	0.95	0.68	0.89 (0.83, 0.94)	24.37	0.87	0.83	0.70
Sum of 3 skinfolds (mm)	0.96 (0.92, 0.99)	68.44	0.92	0.88	0.80	0.82 (0.73, 0.91)	77.20	0.80	0.75	0.55
VO_2_ max (mL/kg/min) ^a^	0.79 (0.69, 0.88)	38.07	0.92	0.67	0.59	0.70 (0.61, 0.80)	35.70	0.83	0.60	0.43
Handgrip right (kg) ^a^	0.56 (0.43, 0.69)	29.40	0.64	0.54	0.18	0.54 (0.42, 0.65)	23.65	0.76	0.47	−0.23
Relative Handgrip right (kg × kg^−1^) ^a^	0.77 (0.67, 0.88)	0.35	0.76	0.79	0.55	0.75 (0.65, 0.84)	0.38	0.79	0.68	0.47
Handgrip left (kg) ^a^	0.55 (0.42, 0.68)	27.95	0.60	0.61	0.21	0.52 (0.40, 0.63)	24.55	0.76	0.41	0.17
Relative Handgrip left (kg × kg^−1^) ^a^	0.77 (0.66, 0.87)	0.34	0.68	0.79	0.47	0.77 (0.68, 0.86)	0.35	0.83	0.75	0.58
CMJ (cm) ^a^	0.77 (0.66, 0.88)	21.55	0.60	0.85	0.45	0.70 (0.59, 0.81)	18.05	0.55	0.79	0.34
Relative CMJ (cm × kg^−1^) ^a^	0.82 (0.76, 0.90)	0.33	0.96	0.74	0.70	0.82 (0.74, 0.90)	0.35	0.90	0.63	0.52
20-m sprint (s)	0.66 (0.53, 0.79)	3.67	0.84	0.48	0.32	0.74 (0.63, 0.84)	4.37	0.60	0.80	0.40
Curl up (reps) ^a^	0.73 (0.61, 0.85)	24.50	0.48	0.92	0.40	0.58 (0.46, 0.70)	17.50	0.41	0.73	0.14
SBJ (m) ^a^	0.75 (0.64, 0.87)	1.41	0.68	0.71	0.39	0.70 (0.60, 0.80)	1.37	0.90	0.49	0.39
Relative SBJ (m × m^−1^) ^a^	0.72 (0.60, 0.84)	0.89	0.84	0.53	0.37	0.67 (0.56, 0.78)	0.83	0.86	0.49	0.35

BMI: body mass index; Corr: corrected; VO_2_ max: maximal oxygen consumption; CMJ: countermovement jump; SBJ: standing broad jump. ^a^ Directionally inverted so that lower physical performance represents higher risk (1–AUC).

**Table 3:** Optimal cut-off, sensitivity, specificity, Youden index and area under the ROC curves for identifying WHtR-defined surrogate cardiometabolic risk in on-time maturing adolescents.

Variables	Males					Females				
	AUC (95% CI)	Cut-Off	Sensitivity	Specificity	Youden	AUC (95% CI)	Cut-Off	Sensitivity	Specificity	Youden
Body mass (kg)	0.75 (0.67, 0.83)	58.05	0.85	0.57	0.42	0.79 (0.69, 0.88)	62.75	0.58	0.89	0.48
BMI (kg/m^2^)	0.96 (0.93, 0.99)	24.75	0.88	0.95	0.82	0.96 (0.92, 0.99)	24.65	0.84	0.94	0.78
Hips girth (cm)	0.90 (0.85, 0.95)	93.55	0.92	0.72	0.64	0.89 (0.81, 0.96)	96.77	0.84	0.81	0.65
Corr. arm girth (cm)	0.74 (0.67, 0.81)	23.12	0.73	0.65	0.38	0.82 (0.72, 0.91)	22.96	0.68	0.90	0.58
Corr. thigh girth (cm)	0.74 (0.66, 0.81)	43.79	0.71	0.63	0.34	0.81 (0.72, 0.90)	43.62	0.68	0.88	0.56
Corr. calf girth (cm)	0.52 (0.43, 0.62)	33.48	0.29	0.83	0.12	0.64 (0.52, 0.75)	29.71	0.55	0.71	0.26
Fat mass (%)	0.91 (0.86, 0.96)	26.28	0.90	0.87	0.77	0.93 (0.89, 0.97)	28.09	0.97	0.78	0.75
Muscle mass (kg)	0.64 (0.56, 0.73)	24.81	0.33	0.88	0.21	0.78 (0.67, 0.88)	17.64	0.77	0.74	0.52
Triceps skinfold (mm)	0.92 (0.89, 0.94)	15.93	0.86	0.84	0.71	0.93 (0.90, 0.96)	22.85	0.82	0.90	0.73
Thigh skinfold (mm)	0.89 (0.86, 0.92)	20.80	0.83	0.82	0.65	0.88 (0.84, 0.92)	29.40	0.85	0.81	0.66
Calf skinfold (mm)	0.92 (0.89, 0.94)	15.99	0.87	0.82	0.70	0.91 (0.87, 0.94)	20.15	0.88	0.82	0.71
Sum of 3 skinfolds (mm)	0.83 (0.76, 0.90)	57.00	0.77	0.82	0.59	0.85 (0.77, 0.93)	71.20	0.81	0.82	0.62
VO_2_ max (mL/kg/min) ^a^	0.69 (0.63, 0.76)	36.71	0.88	0.57	0.45	0.63 (0.54, 0.71)	36.07	0.94	0.34	0.27
Handgrip right (kg) ^a^	0.52 (0.43, 0.60)	23.50	0.81	0.34	0.15	0.59 (0.49, 0.69)	23.15	0.74	0.44	0.18
Relative Handgrip right (kg × kg^−1^) ^a^	0.69 (0.61, 0.77)	0.45	0.73	0.65	0.38	0.68 (0.56, 0.79)	0.33	0.39	0.92	0.31
Handgrip left (kg) ^a^	0.51 (0.43, 0.60)	19.25	0.85	0.21	0.06	0.59 (0.50, 0.69)	21.75	0.71	0.50	0.21
Relative Handgrip left (kg × kg^−1^) ^a^	0.68 (0.60, 0.76)	0.43	0.71	0.63	0.34	0.69 (0.57, 0.80)	0.36	0.61	0.76	0.37
CMJ (cm) ^a^	0.71 (0.62, 0.79)	22.15	0.67	0.72	0.38	0.63 (0.52, 0.75)	15.75	0.36	0.93	0.29
Relative CMJ (cm × kg^−1^) ^a^	0.82 (0.75, 0.89)	0.30	0.67	0.89	0.56	0.76 (0.67, 0.85)	0.28	0.52	0.91	0.43
20-m sprint (s)	0.70 (0.62, 0.79)	4.12	0.58	0.79	0.37	0.62 (0.50, 0.73)	4.28	0.42	0.83	0.25
Curl up (reps) ^a^	0.62 (0.53, 0.71)	29.50	0.56	0.65	0.21	0.57 (0.46, 0.69)	21.50	0.36	0.80	0.15
SBJ (m) ^a^	0.73 (0.65, 0.81)	1.32	0.63	0.78	0.40	0.60 (0.48, 0.73)	1.24	0.55	0.73	0.27
Relative SBJ (m × m^−1^) ^a^	0.69 (0.61, 0.77)	0.84	0.69	0.69	0.38	0.61 (0.49, 0.73)	0.72	0.45	0.85	0.30

BMI: body mass index; Corr: corrected; VO_2_ max: maximal oxygen consumption; CMJ: countermovement jump; SBJ: standing broad jump. ^a^ Directionally inverted so that lower physical performance represents higher risk (1–AUC).

**Table 4:** Optimal cut-off, sensitivity, specificity, Youden index and area under the ROC curves for identifying WHtR-defined surrogate cardiometabolic risk in late-maturing adolescents.

Variables	Males					Females				
	AUC (95% CI)	Cut-Off	Sensitivity	Specificity	Youden	AUC (95% CI)	Cut-Off	Sensitivity	Specificity	Youden
Body mass (kg)	0.75 (0.56, 0.93)	56.27	0.64	0.70	0.33	0.74 (0.53, 0.94)	53.92	0.88	0.57	0.44
BMI (kg/m^2^)	0.95 (0.91, 1.00)	22.50	0.91	0.87	0.77	0.99 (0.97, 1.00)	24.78	1.00	0.91	0.91
Hips girth (cm)	0.90 (0.81, 0.99)	93.05	0.82	0.83	0.65	0.97 (0.93, 1.00)	97.97	1.00	0.86	0.86
Corr. arm girth (cm)	0.72 (0.52, 0.91)	23.03	0.64	0.84	0.48	0.77 (0.60, 0.93)	21.32	0.88	0.68	0.55
Corr. thigh girth (cm)	0.73 (0.54, 0.93)	42.97	0.55	0.98	0.52	0.80 (0.63, 0.97)	41.22	0.75	0.75	0.50
Corr. calf girth (cm)	0.65 (0.46, 0.83)	32.19	0.55	0.87	0.41	0.64 (0.38, 0.91)	31.63	0.50	0.96	0.46
Fat mass (%)	0.88 (0.80, 0.97)	24.04	0.82	0.83	0.65	0.88 (0.76, 1.00)	28.02	0.75	0.94	0.69
Muscle mass (kg)	0.69 (0.48, 0.90)	24.69	0.64	0.85	0.49	0.78 (0.62, 0.95)	15.97	1.00	0.49	0.51
Triceps skinfold (mm)	0.85 (0.77, 0.92)	14.32	0.78	0.79	0.58	0.90 (0.82, 0.99)	23.80	0.83	0.89	0.72
Thigh skinfold (mm)	0.80 (0.70, 0.90)	19.15	0.78	0.77	0.56	0.80 (0.68, 0.92)	31.80	0.78	0.76	0.54
Calf skinfold (mm)	0.85 (0.77, 0.93)	15.80	0.74	0.87	0.61	0.90 (0.85, 0.96)	23.60	0.83	0.85	0.68
Sum of 3 skinfolds (mm)	0.82 (0.69, 0.96)	52.00	0.82	0.82	0.64	0.82 (0.62, 0.92)	70.09	0.83	0.85	0.67
VO_2_ max (mL/kg/min) ^a^	0.72 (0.59, 0.84)	37.49	1.00	0.67	0.59	0.58 (0.41, 0.75)	24.77	0.75	0.59	0.34
Handgrip right (kg) ^a^	0.63 (0.46, 0.80)	27.20	0.73	0.58	0.31	0.66 (0.45, 0.86)	27.25	0.75	0.64	0.39
Relative Handgrip right (kg × kg^−1^) ^a^	0.57 (0.36, 0.78)	0.41	0.46	0.86	0.31	0.57 (0.30, 0.84)	0.40	0.63	0.73	0.35
Handgrip left (kg) ^a^	0.66 (0.48, 0.83)	28.90	0.64	0.71	0.35	0.66 (0.45, 0.87)	24.55	0.75	0.67	0.42
Relative Handgrip left (kg × kg^−1^) ^a^	0.54 (0.32, 0.75)	0.40	0.46	0.81	0.27	0.55 (0.29, 0.81)	0.37	0.63	0.72	0.34
CMJ (cm) ^a^	0.55 (0.36, 0.73)	28.85	0.54	0.64	0.18	0.75 (0.52, 0.97)	17.95	0.75	0.81	0.56
Relative CMJ (cm × kg^−1^) ^a^	0.73 (0.58, 0.87)	0.46	0.82	0.68	0.50	0.77 (0.54, 1.00)	0.32	0.75	0.86	0.61
20-m sprint (s)	0.56 (0.39, 0.72)	3.90	0.91	0.20	0.11	0.58 (0.32, 0.85)	4.40	0.50	0.82	0.32
Curl up (reps) ^a^	0.56 (0.42, 0.70)	34.50	0.82	0.42	0.24	0.65 (0.43, 0.87)	23.50	0.63	0.69	0.32
SBJ (m) ^a^	0.59 (0.42, 0.76)	1.26	0.54	0.68	0.22	0.67 (0.41, 0.93)	1.21	0.75	0.69	0.44
Relative SBJ (m × m^−1^) ^a^	0.59 (0.42, 0.77)	0.98	0.64	0.60	0.24	0.67 (0.43, 0.92)	0.69	0.63	0.81	0.43

BMI: body mass index; Corr: corrected; VO_2_ max: maximal oxygen consumption; CMJ: countermovement jump; SBJ: standing broad jump. ^a^ Directionally inverted so that lower physical performance represents higher risk (1–AUC).

## Data Availability

The datasets generated during and/or analysed during the current study are available from the corresponding author on reasonable request.

## Supplementary Materials

The following supporting information can be downloaded at: https://www.mdpi.com/article/10.3390/children13091201/s1. Supplementary Table S1. Internal bootstrap validation (1000 resamples) for early maturers; Supplementary Table S2. Internal bootstrap validation (1000 resamples) for on-time maturers; Supplementary Table S3. Internal bootstrap validation (1000 resamples) for late maturers; Supplementary Figure S1. Complete receiver operating characteristic (ROC) curves for all evaluated anthropometric, body composition, and physical fitness variables in discriminating WHtR-defined cardiometabolic risk across biological maturation groups and sexes.
