# Supplementary material for: Discriminatory Capacity of Anthropometric Indicators and Physical Fitness Tests for Identifying Waist-to-Height Ratio-Defined Cardiometabolic Risk in Adolescents According to Sex and Biological Maturity
Source: Children (Basel). 2026 Sep 5;13(9):1201. doi: 10.3390/children13091201 (PMC13605130; doi:10.3390/children13091201)
Supplement: Supplementary file 1 [file children-13-01201-s001.zip › children-4523093-supplementary.pdf]

Supplementary Table S1. Internal bootstrap validation (1000 resamples) for early maturers.

Variable	Males				Females			
	Estimate	Bias	SE	95% CI	Estimate	Bias	SE	95% CI
Body mass (kg)	0.264	0.07	0.068	0.07, 0.34	0.021	-0.17	0.043	-0.47, 0.16
BMI (kg/m^2)	1.206	0.27	0.403	-0.01, 1.57	1.884	0.34	0.416	0.14, 2.95
Hips girth (cm)	0.257	0.88	0.137	-0.11, 0.42	0.185	0.05	0.138	-0.28, 0.85
Corr. arm girth (cm)	2.717	0.35	0.576	0.65, 2.90	0.609	0.06	0.346	-1.12, 1.89
Corr. thigh girth (cm)	1.430	0.29	0.398	0.30, 1.86	0.233	-0.16	0.210	-0.75, 1.30
Corr. calf girth (cm)	0.565	0.07	0.276	-0.17, 0.92	-0.746	-0.18	0.285	-1.61, 0.32
Fat mass (%)	0.038	0.33	0.105	-0.20, 0.22	-0.354	-0.08	0.110	-0.82, 0.10
Muscle mass (kg)	-3.946	-0.06	0.783	-4.31, -1.24	-1.262	-0.11	0.486	-2.64, 2.00
Triceps skinfold (mm)	0.414	0.34	0.222	-0.02, 0.85	-0.034	-0.07	0.121	-0.27, 0.20
Thigh skinfold (mm)	0.086	0.10	0.144	-0.20, 0.37	0.029	0.03	0.072	-0.11, 0.17
Calf skinfold (mm)	-0.013	-0.28	0.163	-0.33, 0.31	0.430	0.09	0.181	0.08, 0.78
Sum of 3 skinfolds (mm)	0.052	0.06	0.070	-0.09, 0.19	-0.049	0.04	0.047	-0.14, 0.04
VO_2 max (ml/kg/min)	0.015	-0.09	0.150	-0.28, 0.31	0.392	0.04	0.236	-0.07, 0.86
Handgrip right (kg)	-0.076	-0.30	2.802	-5.57, 5.42	-0.325	-0.10	0.641	-1.58, 0.93
Relative Handgrip right (kg × kg^{-1})	1.217	0.08	2.056	-3.90, 4.15	-2.857	0.29	1.833	-5.60, 1.88
Handgrip left (kg)	0.011	0.39	2.578	-5.04, 5.06	-0.117	0.32	0.568	-1.23, 1.00
Relative Handgrip left (kg × kg^{-1})	-3.657	-0.12	1.894	-5.75, 5.68	2.473	0.05	1.723	-1.85, 5.17
CMJ (cm)	0.030	-0.27	0.638	-1.22, 1.28	-0.145	-0.25	0.337	-0.61, 0.52
Relative CMJ (cm × kg^{-1})	1.533	0.19	0.566	-2.09, 2.12	5.611	0.30	2.269	1.89, 8.09
20-m sprint (s)	-1.286	-0.52	1.486	-2.61, 2.51	1.315	0.12	1.294	-1.22, 3.85
Curl up (reps)	0.180	0.89	0.126	-0.07, 0.43	0.071	0.78	0.039	-0.01, 0.15
SBJ (m)	1.994	0.28	1.620	-2.82, 2.69	2.674	0.18	1.967	1.81, 5.30
Relative SBJ (m × m^{-1})	-2.597	-0.40	2.675	-5.84, 2.65	-3.007	-0.11	1.148	-5.27, -0.83

SE: standard error; CI: confidence interval. Internal validation was performed using a non-parametric bootstrap procedure with 1,000 resamples and bias-corrected and accelerated (BCa) 95% confidence intervals. Dependent variable: WHtR-defined cardiovascular/cardiometabolic risk.

Supplementary Table S2. Internal bootstrap validation (1000 resamples) for on-time maturers.

Variable	Males				Females			
	**Estimate**	**Bias**	**SE**	**95% CI**	**Estimate**	**Bias**	**SE**	**95% CI**
Body mass (kg)	0.031	0.01	0.05	-0.09, 0.12	-0.080	-0.01	0.044	-0.14, 0.03
BMI (kg/m^2)	1.419	0.15	0.33	0.82, 1.89	1.468	0.14	0.289	0.93, 1.88
Hips girth (cm)	0.093	0.03	0.10	-0.13, 0.25	-0.155	0.02	0.085	-0.29, 0.03
Corr. arm girth (cm)	0.581	0.11	0.31	-0.18, 1.10	-0.208	-0.01	0.291	-0.87, 0.27
Corr. thigh girth (cm)	0.704	0.04	0.24	0.23, 1.13	-0.004	0.01	0.232	-0.50, 0.46
Corr. calf girth (cm)	-0.210	-0.01	0.17	-0.52, 0.19	-0.110	-0.08	0.184	-0.43, 0.17
Fat mass (%)	-0.063	-0.02	0.05	-0.14, 0.06	-0.025	-0.03	0.063	-0.09, 0.15
Muscle mass (kg)	-1.462	-0.20	0.44	-1.99, -0.09	0.120	-0.02	0.392	-0.61, 1.01
Triceps skinfold (mm)	0.396	-0.07	0.061	0.08, 0.67	0.278	0.34	0.081	0.03, 0.35
Thigh skinfold (mm)	-0.120	0.31	0.264	-0.31, 0.06	0.038	0.06	0.125	-0.25, 0.20
Calf skinfold (mm)	0.199	-0.09	0.083	-0.05, 0.49	0.281	0.19	0.129	-0.04, 0.33
Sum of 3 skinfolds (mm)	-0.018	0.04	0.011	-0.12, 0.05	-0.074	-0.08	0.268	-0.13, 0.89
VO_2 max (ml/kg/min)	0.008	-0.05	0.130	-0.40, 0.13	0.030	0.01	0.102	-0.21, 0.29
Handgrip right (kg)	-0.124	0.29	1.107	-2.27, 1.95	-1.117	-0.08	1.285	-2.57, 2.13
Relative Handgrip right (kg × kg^{-1})	2.623	-0.41	0.639	-1.97, 4.66	0.684	-0.05	0.800	-1.18, 1.50
Handgrip left (kg)	-0.078	0.02	1.011	-1.15, 1.84	1.619	0.14	1.546	-1.22, 3.73
Relative Handgrip left (kg × kg^{-1})	2.693	0.22	0.732	-1.27, 5.81	-0.964	-0.08	0.951	-2.68, 0.95
CMJ (cm)	-0.306	-0.04	0.688	-2.47, 0.28	-0.953	-0.11	0.967	-1.26, 0.38
Relative CMJ (cm × kg^{-1})	1.518	0.28	0.606	-1.02, 3.79	0.519	0.09	0.521	-2.39, 3.62
20-m sprint (s)	-0.380	-0.05	0.166	-2.38, 1.11	-0.166	0.08	0.185	-1.99, 2.06
Curl up (reps)	0.044	0.09	0.018	-0.01, 0.16	0.022	0.02	0.033	-0.06, 0.10
SBJ (m)	2.358	0.18	0.342	1.36, 3.55	1.868	0.16	1.584	-1.34, 3.65
Relative SBJ (m × m^{-1})	-3.610	-0.32	0.597	-5.67, -1.73	-2.932	-0.27	2.571	-5.15, 0.52

SE: standard error; CI: confidence interval. Internal validation was performed using a non-parametric bootstrap procedure with 1,000 resamples and bias-corrected and accelerated (BCa) 95% confidence intervals. Dependent variable: WHtR-defined cardiovascular/cardiometabolic risk.

Supplementary Table S3. Internal bootstrap validation (1000 resamples) for late maturers.

Variable	Males				Females			
	Estimate	Bias	SE	95% CI	Estimate	Bias	SE	95% CI
Body mass (kg)	0.077	-0.18	0.082	-0.02, 0.15	-0.628	-0.25	0.878	-1.52, 0.31
BMI (kg/m^2)	1.342	-0.09	0.925	-0.52, 2.15	0.799	0.17	0.881	-1.98, 1.17
Hips girth (cm)	-0.177	0.13	0.083	-0.50, 0.11	1.432	0.16	1.170	-1.24, 2.73
Corr. arm girth (cm)	0.235	0.04	0.152	-0.43, 0.47	0.151	0.34	0.655	-0.75, 0.94
Corr. thigh girth (cm)	0.418	-0.07	0.593	-0.23, 0.98	-0.287	-0.17	0.498	-0.63, 0.28
Corr. calf girth (cm)	-0.016	-0.04	0.156	-0.19, 0.18	-0.717	-0.25	0.903	-1.53, 0.22
Fat mass (%)	-0.027	-0.02	0.051	-0.07, 0.09	-0.476	-0.06	0.605	-1.07, 0.31
Muscle mass (kg)	-1.340	0.34	0.903	-1.56, 0.37	-0.690	-0.30	0.928	-1.73, 0.32
Triceps skinfold (mm)	0.355	-0.35	0.511	-0.21, 0.98	2.251	0.11	0.932	0.42, 4.08
Thigh skinfold (mm)	0.346	0.04	0.426	-0.05, 0.85	1.430	0.17	0.542	0.37, 2.49
Calf skinfold (mm)	0.407	0.14	0.597	-0.21, 1.01	2.095	0.08	0.944	0.24, 3.95
Sum of 3 skinfolds (mm)	-0.709	-0.04	0.887	-1.52, 0.17	-1.043	-0.05	0.844	-2.70, 0.61
VO_2 max (ml/kg/min)	2.439	0.13	1.347	-0.34, 3.79	-1.993	-0.16	0.705	-3.37, -0.61
Handgrip right (kg)	0.638	-0.21	0.408	-0.21, 1.17	-0.547	-0.07	0.234	-1.01, -0.09
Relative Handgrip right (kg × kg^{-1})	2.949	0.24	1.963	-0.46, 3.98	3.129	0.34	1.086	1.00, 5.26
Handgrip left (kg)	-0.355	0.16	0.467	-0.91, 0.24	-0.309	0.17	0.251	-0.80, 0.18
Relative Handgrip left (kg × kg^{-1})	3.488	-0.17	1.698	-0.73, 3.71	3.718	-0.06	1.290	1.19, 6.25
CMJ (cm)	0.219	-0.03	0.228	-0.03, 0.57	0.250	0.07	0.228	-0.20, 0.70
Relative CMJ (cm × kg^{-1})	0.645	0.08	0.439	-0.26, 1.43	-0.972	-0.04	0.630	-2.21, 0.26
20-m sprint (s)	-0.653	-0.18	0.425	-1.29, 0.35	0.366	0.09	0.282	-0.19, 0.92
Curl up (reps)	0.668	0.03	0.484	-0.87, 1.52	0.209	-0.28	0.087	0.04, 0.38
SBJ (m)	2.233	0.11	1.224	-0.95, 3.47	-3.292	-0.10	1.032	-5.31, -1.27
Relative SBJ (m × m^{-1})	-1.530	-0.24	2.401	-3.71, 1.46	0.484	0.15	0.247	0.00, 0.97

SE: standard error; CI: confidence interval. Internal validation was performed using a non-parametric bootstrap procedure with 1,000 resamples and bias-corrected and accelerated (BCa) 95% confidence intervals. Dependent variable: WHtR-defined cardiovascular/cardiometabolic risk.

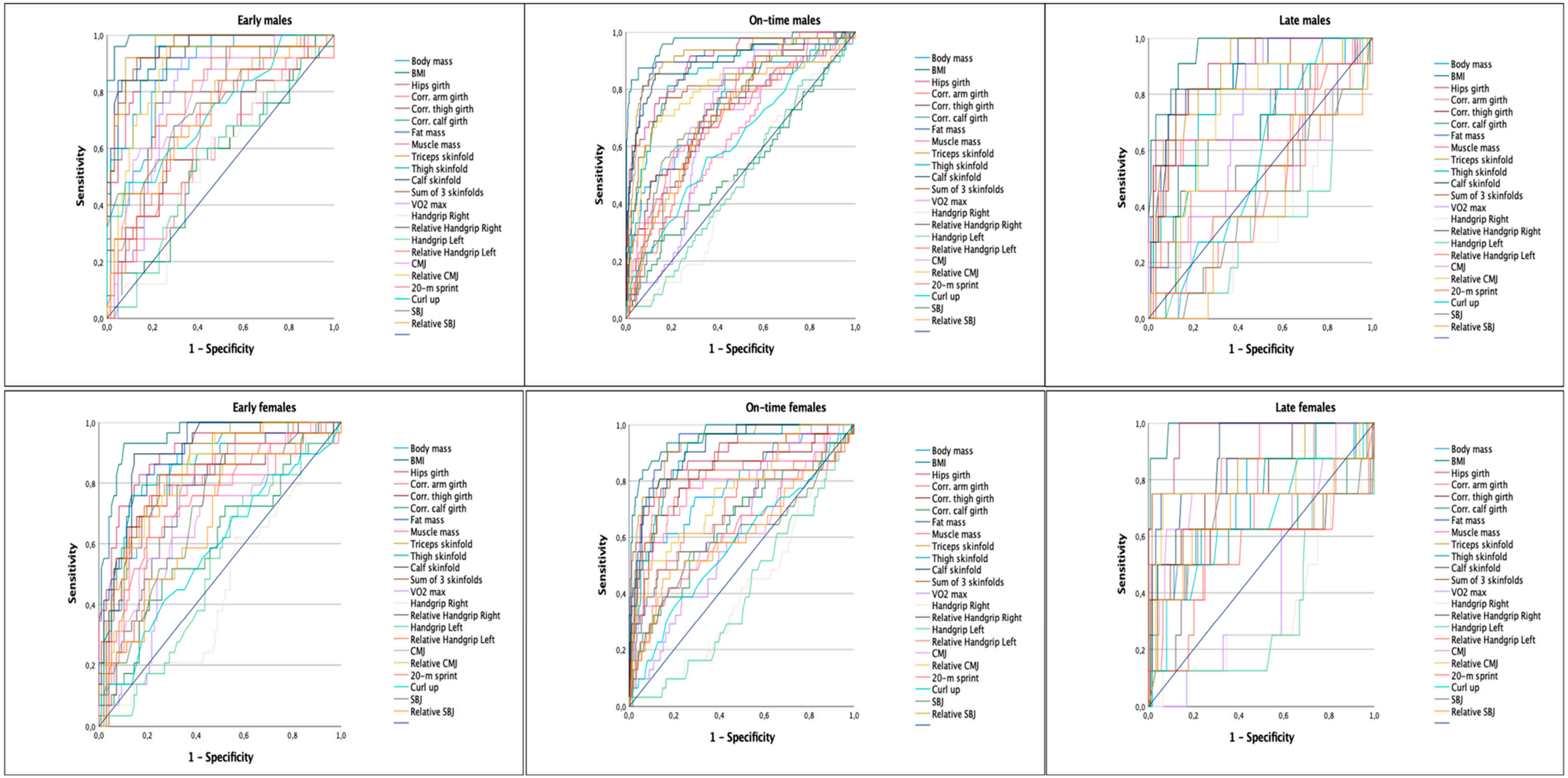

Supplementary Figure S1. Complete receiver operating characteristic (ROC) curves for all evaluated anthropometric, body composition, and physical fitness variables in discriminating WHtR-defined cardiometabolic risk across biological maturation groups and sexes.
